# ElectromyoFigureic Evaluation of Functional Adaptation of Patients with New Complete Dentures

**DOI:** 10.1155/2018/2412084

**Published:** 2018-03-11

**Authors:** Kujtim Sh. Shala, Linda J. Dula, Teuta Pustina-Krasniqi, Teuta Bicaj, Enis F. Ahmedi, Zana Lila-Krasniqi, Arlinda Tmava-Dragusha

**Affiliations:** Department of Prosthetic Dentistry, School of Dentistry, Faculty of Medicine, University of Prishtina, Prishtina, Kosovo

## Abstract

**Objective:**

The objective of this study was to evaluate the level of adaptation of patients to newly fitted complete dentures in their dominant and nondominant sides, by means of ElectromyoFigureic signals.

**Materials and Methods:**

Eighty-eight patients with complete dentures were evaluated in the study. Masticatory muscle (*masseter and temporal*) bioelectric activity of the patients with complete dentures was recorded at maximum intercuspal relation. Parametric statistical data were analyzed with one-way repeated measures ANOVA test.

**Results:**

Measurement time was significantly different for both dominant (DS) and nondominant (NDS) sides: FΣs-DS = 21.51, *p*=0.0001; FΣs-NDS = 13.25, *p*=0.0001. Gender was also significantly different: FΣs-DS-gender = 41.53, *p*=0.001; FΣs-NDS-gender = 85.76, *p*=0.0001. The average surface area values showed significant difference in females. Prior experience with dentures showed no significant difference for both sides of mastication: FΣs-DS-experiences = 1.83, *p*=0.1772; F Σs-NDS-experiences = 3.30, *p*=0.0697.

**Conclusion:**

The planimetric indicators of bioelectric activity of *masseter* and *temporalis* muscles at maximum physiological loading conditions are significant discriminators of the level of functional adaptation of patients with new complete dentures.

## 1. Introduction

Tooth loss and the loss of periodontal afferent flow lead to changes in the masticatory neuromuscular patterns [1]. The ElectromyoFigureic (EMG) tests of *masseter* and *temporalis* muscles are utilized to determine the correlation between electromyoFigureic activity of masticatory muscles and occlusal relations [2–3], craniofacial morphology [4, 5], and various therapeutic procedures [6, 7].

ElectromyoFigurey (EMG) is defined as the Figureic recording of the electrical potential of muscle's performance and their interrelation, based on the analysis of electrical signals produced during each muscle contraction [8]. It has been the only tool for the assessment of muscle activity of stomatognathic system since its first concerted use in dentistry by Moyers in 1949 [3]. Studies by Kemsley et al. [9] and Hagberg [10] have shown that *masseter* and *temporalis* muscles are preferred in EMG studies of masticatory function. Clinicians and researchers have historically used EMG to test the masticatory function of denture wearers.

EMG research suggests that the overall activity of mandibular elevator muscles in denture wearers does not significantly differ from patients with natural dentition [11]. However, there is no clear explanation as to why tooth loss reduces the capacity of masticatory muscles to perform [12]. In other words, as a result of tooth loss, although muscle activity is maintained, there is a significant reduction of masticatory efficiency in patients with complete dentures. The latter occurs due to the lack of adequate masticatory muscle activity (i.e., loss of periodontal receptors) and altered energy distribution within masticatory muscles. Pancherz [13] analyzed the integrated activity of *masseter* and *temporalis* muscles, with the help of standard electrode techniques, and compared the results between experimental homologous groups. Quantitative analysis of EMG activity between *masseter* and *temporalis* muscles was tested in relation to the maximum bite forces in the intercuspidal position and in relation to the masticatory cycle obtained during the crushing of a peanut grain. The results obtained in this research have shown that EMG activity of *masseter* muscle increases over time at maximum bite force and during mastication, while *temporal* muscle activity remains relatively constant. These results are consistent with the results of other human [2] and animal [14] studies.

One of the objectives of prosthetic rehabilitation is to ensure the best possible masticatory function of the patient.

## 2. Main Hypothesis

This study is part of a wide research with the following working hypothesis: “Optimum period of functional stability in Complete Denture Wearers, following the completion of the period of neuromuscular adjustment.” The hypothesis may be determined with the help of the following functional tests:

- Interocclusal perception test
- Maximum mastictory load test
- Masticatory muscle bioelectric activity test
- Masticatory efficiency test

The aim of this study was to evaluate the level of patients' adaptation in experienced and nonexperienced denture wearers after the insertion of new complete dentures in dominant side (DS) and nondominant side (NDS). To do so, we used ElectromyoFigureic signals for a period of six months after fitting the dentures.

## 3. Materials and Methods

The research proposal was accepted and approved by the Ethics Committee of the University Dentistry Clinical Center of Kosovo, Prishtina. Informed consent was obtained from each individual participant in this study.

Eighty-eight patients with complete dentures with eugnatic jaws in sagittal plain, and no signs of orofacial system dysfunction were examined. There were 42 females and 46 males. Forty-five patients belonged to the nonexperienced group which wore new complete dentures for the first time, while forty-three patients had been wearing complete dentures for a while (the experienced group) (Table 1).

All the examinees were subject to history taking and clinical examination of dominant masticatory side in function. All examinees have no signs of orofacial conditions.

Patients over 70 years of age with orthodontic anomalies in sagittal and transversal planes, dentofacial conditions, and significant resorbtion of alveolar ridge (i.e., negative alverolar ridge) were excluded from the study. Depending on their experience with denture wearing, the sample was divided into two groups:

- Group 1: patients fitted with dentures for the first time nonexperienced—complete denture wearers (nCDW)
- Group 2: patients who had had previous experience with complete dentures—experienced complete denture wearers (eCDW)

The (convenient) random sample of patients was selected at the Department of Prosthodontics, University of Prishtina, Faculty of Medicine, Stomatology Branch, Prishtina, Kosovo.

In order to evaluate the masticatory muscle activity of CDW, EMG bioelectric activity of *masseter* and *temporalis* muscles was recorded, unilaterally, at maximum bite force (mBF). The successive measurement (observational) periods were at first week of fitting the complete dentures and five weeks, ten weeks, 15 weeks, 20 weeks, and 25 weeks after fitting the complete dentures.

During signal acquisition, the subject remained comfortably seated in a chair and was presented with the equipment and the movements to be performed, getting all the necessary instructions and information. EMG recording was carried out with the DynoFigureic Quadrant R 511 A, with the possibility of direct and integrated recording. The action potential was measured via bipolar electrodes. The electrodes were placed in the patients' auricle or hand. Considering that a significant number of measurements are required at the time of observation, the constant position of the electrode was ensured. Specifically, a plastic template which contained the pressure of points of *masseter* and *temoralis* muscles in a coordinate system was used for each measurement. Within the earlier mentioned measurement (observation) periods, three consecutive mBF measurements were conducted lasting three seconds each with intermittent pauses of one minute of muscular relaxation time at the dominant side (DS) first and nondominant side (NDS) last. During initial isometric contraction time on clenching, in central relation (maximum intercuspal position), muscular tonus increased.

EMG recorded the amplitude and the sum of surfaces of the planimetric indicators of the bioelectric activity of *masseter* and *temporalis* muscles. The surface of the planimetric indicators of the bioelectric activities of *masseter* and *temporalis* muscles was measured with the aid of Reiss number 3005 and is presented as the mean value between the two measurements. The sum of the surfaces of planimetric indicators of bioelectric activity of *masseter* and *temporalis* muscles was obtained by the sum of constituent components (Σ*P* = Pmm + Pmt). For the analysis, the highest obtained value of the surface of planimetric indicators and bioelectric activity of *masseter* muscle was obtained at earlier defined time intervals. The corresponding sum of surfaces of the planimetric indicators of bioelectric activity of *temporalis* muscle was added to the latter value. Within the scope of this scheme of bioelectric activities of *masseter* and *temporalis* muscles, maximal amplitude of each individual muscle was calculated and used in the analysis of the results.

## 4. Data Analysis

Statistical analysis was performed using the standard software package BMDP (Biomedical Statistical Package), dedicated to research in the biomedical sciences and including all methods of statistical procedures (Dixon, 62). Testing parametric data was done with one-way repeated measures ANOVA test.

## 5. Results

The tables present the average values of the electromyoFigureic activity of *masseter* and *temporalis* muscles in dominant side and nondominant side during maximum voluntary tooth compression at the intercuspidal position. The results presented in the tables are elaborated in the following:

1. *The amplitude dynamics of the planimetric indicators of bioelectric activity of m. masseter during maximum voluntary tooth compression at the intercuspidal position presented with these values*: by analysis of variation, the influence of the time of measurement in the change of the amplitude of m. masseter was more significant in dominant side (DS) compared to the nondominant side (NDS): Fa.m.m.-DS = 16.06, *p*=0.0001; Fa.m.m.-NDS = 9.39, *p*=0.0001.

In DS, the amplitude of the m. masseter increased steadily from the initial values to the third measurement. However, it marked a collapse in the fourth measurement but then reached maximum value again, which it sustained throughout the fifth and sixth measurements. In NDS, the value of the amplitude conveys the same dynamics but with less pronounced changes. In addition, the stationary state (when there is no muscular contraction activity) is reached after the fourth measurement (Table 2).

**Figure 1:**
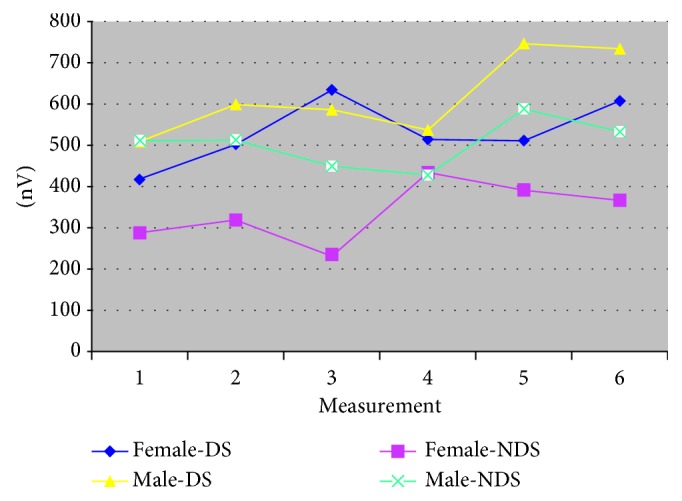
The amplitude of m. masseter according to gender with complete dentures (dominant side/nondominant side).

Gender influences the average values of the amplitude of m. masseter: Fa.m.m-DS-gender = 34.4, *p*=0.0001; Fa.m.m-NDS-gender = 239.59; *p*=0.001. In addition, gender interacts with the time of measurement to lead to different results. Fa.m.m-DS-interaction = 7.06, *p*=0.0001; Fa.m.m-NDS-interactions = 10.65, *p*=0.0001 (Table 2 and Figure 1).

**Figure 2:**
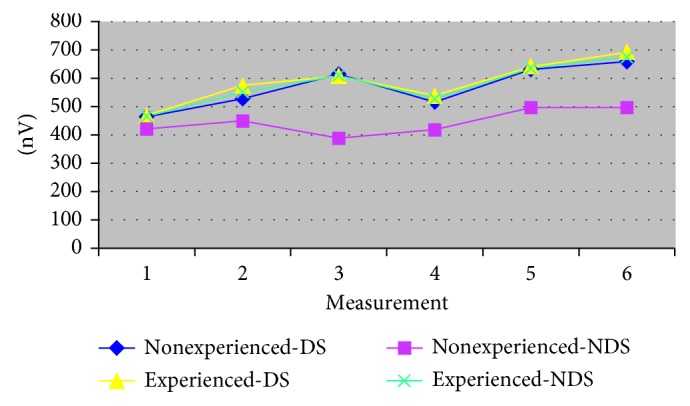
The amplitude of m. masseter according to experience with complete dentures (dominant side/nondominant side).

The amplitude values of m. masseter in the DS and the NDS do not vary depending on the variables of the previous experience with complete dentures. Fa.m.m.-experience DS = 1.25, *p*=0.2648; Fa.m.m.-experience NDS = 3.23, *p*=0.0729 (Table 2 and Figure 2).

(2) *The amplitude dynamics of the planimetric indicators of bioelectric activity of m. temporalis during maximum voluntary tooth compression at the intercuspidal position presented with these values*: the average values of m. temporalis amplitude have significant differences only on the dominant side (DS) compared to the nondominant side (NDS): Fa m.t-DS = 3.28, *p*=0.0063; Fa m.t-NDS = 1.62, *p*=0.1518. While the amplitude values in the first three measurements are approximately the same, in the fourth measurement, there is a noticeable reduction of the amplitude values. Further, in the last two measurements, they approach the initial values again. Analysis suggests that there is no reaction in NDS, except for the natural variation between the values of the amplitude between successive measurements (Table 3).

**Figure 3:**
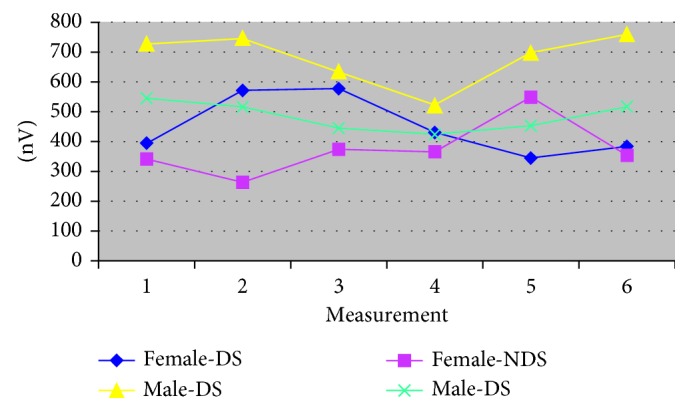
The amplitude of m. temporalis according to gender with complete dentures (dominant side/nondominant side).

**Figure 4:**
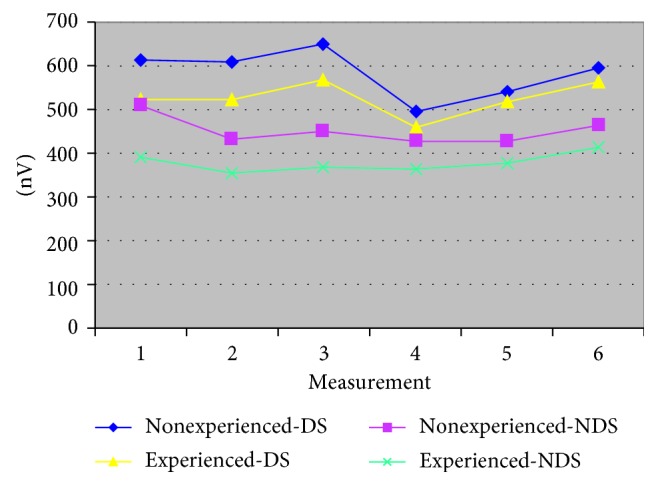
The amplitude of m. temporalis according to experience with complete dentures (dominant side/nondominant side).

The gender of the researchers and the interaction of time with gender affect the change of the values of the amplitude of m. temporalis: Fa m.t-DS-gender = 282.6, *p*=0.0001; Fa m.t-NDS-gender = 112.63, *p*=0.0001; Fa m.t-NDS-interaction = 5.60, *p*=0.0001 (Table 3 and Figure 3). Significant differences in the amplitude of the m. temporal also occur as a result of the influence of the prior experience in both DS and NDS: Fa m.t-DS-experience = 7.81 *p*=0.0054; Fa m.t-NDS-experiences = 25.07, *p*=0.0001 (Table 3 and Figure 4).

(3) *The values of the sum of the surfaces of the planimetric indicators of the bioelectric activity of m. masseter and m. temporalis presented with these values*: the influence of the measurement time on the values of the amount of surfaces is important both in DS and in NDS: FΣs-DS = 21.51, *p*=0.0001; FΣs-NDS = 13.25, *p*=0.0001. The initial values of DS amounts are lower than the others. The stationary is measured in the fifth measurement, suggesting that, in the NDS, the first four measurements did not differ, but in the fifth and sixth measurements, there were approximate values of the sum of the surfaces (Table 4).

**Figure 5:**
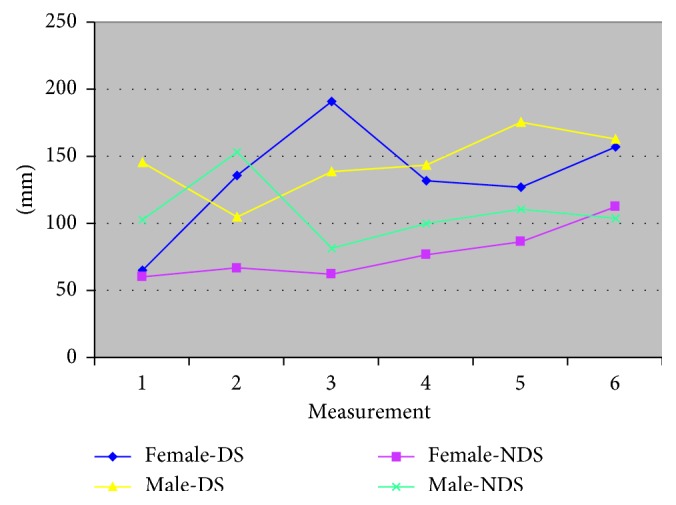
The values of the sum of the surfaces of the planimetric indicators according to gender with complete dentures (dominant side/nondominant side).

The gender impact is of great importance on both sides and in the interaction with the time of measurement: FΣs-DS-gender = 41.53, *p*=0.001; FΣs-NDS-gender = 85.76, *p*=0.0001; FΣs-DS-interactions = 34.6, *p*=0.001; and FΣs-NDS-interactions = 8.37, *p*=0.0001. The average value of the surface area has significant differences in females and rose with the time of measurement (Figure 5).

**Figure 6:**
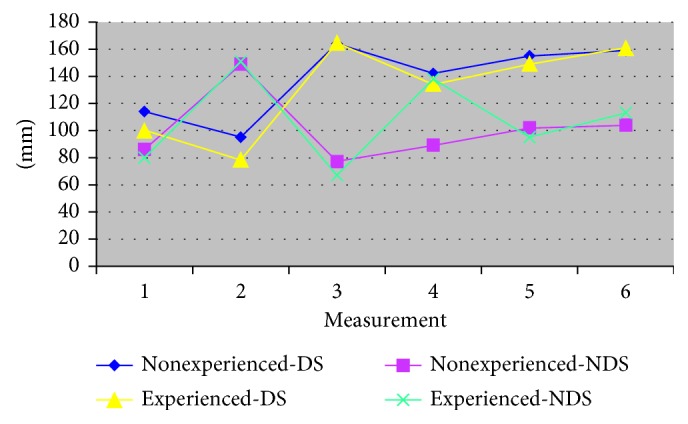
The values of the sum of the surfaces of the planimetric indicators according to experience.

The impact of the preliminary experience with the complete dentures did not matter either in DS and NDS: FΣs-DS-experiences = 1.83, *p*=0.1772; FΣs-NDS-experiences = 3.30, *p*=0.0697 (Figure 6).

## 6. Discussion

This study investigated the behavior of EMG parameters during maximal voluntary tooth compression in the intercuspidal position. We paid special attention to how the main mandibular elevator, under maximum load conditions, expressed their activity in the intercuspidal position. This is the activity that affects the dynamics of functional adaptation to the new complete dentures. New prostheses have positive effect on the patient's muscular activity. However, an adaptation period of the muscle fibers to the new prosthesis is needed [15]. Goiato et al. concluded that a new complete denture allows for neuromuscular reprogramming, which contributes to muscular balance of the masticatory system [16]. Moller and Hannam et al. have proved that the stability of the intercuspidal position is of great clinical significance because this position can generate large masticatory forces, as the muscular activity in this position is maximally expressed [5, 17]. During the period of initial isometric contraction in conditions of tightness of the teeth in the maximal intercuspidation, there is an increase in muscle tonus. This increase is in a positive linear relationship with the number of planes of EMG (quantitative parameter) of the muscles involved [18].

The maximum amplitude within the planimetric indicators is of qualitative importance as it is the result of the bioelectric activity of the recruited motor units during the generation of a masticatory force. The planimetric indicators were analyzed separately in relation to the dynamics of the component: the amount of surfaces and the amplitude of the bioelectric activity of m. masseter and m. temporalis during under maximum load conditions. The amplitude average values of m. masseter and m. temporalis during the maximum voluntary tooth compression are about 1/3 lower in the DS than in NDS, while in the NDS, it is detected at several defined time intervals. No significant difference was found in the amplitude values of m. masseter and m. temporalis during maximum voluntary tooth compression at the intercuspidal position between DS and NDS. Edentulous subjects also produced significantly less EMG activity and had significantly lower estimated jaw muscle strength. Weakened jaw muscles are one factor contributing to lower maximum bite forces among users of conventional dentures [19]. A previous study of elevator muscle activity in patients before and after complete dentures suggested that the use of complete dentures provokes electromyoFigureic changes by increasing the occlusal vertical dimension [20]. The behavior of the research determines functional adaptation to new complete denture, and it oscillates around the balancing position. This result is also supported by Karakazis and Kossion, who reported that the chewing efficiency showed a noticeable increase with time. This is because improving denture adaptation may be due to the neuromuscular control which is gradually generated by time [21].

Our findings suggest that there is a difference due to gender, thus aligning with previous research. Gender influences the average values of the amplitude of m. masseter. According to previous research, females have lower amplitude of m. masseter compared to male in DS and NDS. With the increase in the number of measurements, these values rise, but in the fourth measurement, there is a decrease in values. In female participants, the average amplitude of m. temporalis is significantly lower than that of male participants. The dynamic changes of the amplitude values of m. temporal occur at different periods. Additionally, the values of the amount of planimetric surface areas are significantly lower in females compared to males. This is in consistent with the previous findings, which revealed a significant gender difference in masticatory performance, and suggests that the greater muscular potential of the males may be attributed to the anatomic differences [22–24]. The masseter muscles of males have type 2 fibers with larger diameter and a greater sectional area than those of the females [25].

The experience of patient's wearing complete dentures is the most important factor in which intensive reactions are observed during the observational period. The amplitude values of m. masseter in DS and the NDS do not vary depending on the variables of the previous experience complete dentures. On the contrary, patients with no experience with complete denture have higher amplitude of m. temporalis compared to experienced patients. This report confirms Moller's opinion that m. temporalis is the main postural mandibular muscle [26]. Electrical activity during tooth clenching exhibited a statistically significant reduction only in the right m. temporal after five months of wearing the new complete dentures [18]. It is interesting that previous experience has not been shown as a factor which affects change in the value of the surface area of the planimetric indicators on both the dominant side and the nondominant side. These observations confirm that wavelet-based EMG analysis is instrumental in evaluating denture adaptation for patients with complete dentures replacement, and denture adaptation increases with time [27].

The fact is that some studies observed differences in the proportion of fiber types between denture wearers, and dentate subjects cannot be ascribed to degenerative changes intrinsic to the ageing muscle. Instead, this is caused by functional differences in muscle activity and morphological alterations of stomatognathic system accompanying the complete teeth loss [28]. Tooth loss and use of complete dentures affect the motor and sensorial aspects involved in the masticatory process. Information received centrally is not sufficiently accurate to allow adaptation of mastication patterns to the food texture in denture wearers [29]. Treatment by implant-supported oral rehabilitation in the elderly individuals revealed a decrease in electromyoFigureic amplitude for the masseter muscles during swallowing of pasty and liquid foods [30]. Edentulous patients with implant-supported fixed dental prostheses are a very invasive, expensive, long treatment option but at the same time a valuable treatment option for restoring edentulous patients [31]. These data will allow clinicians to objectively make clinical decisions and predict future treatment outcomes.

## 7. Conclusions

Components of planimetric indicators of bioelectric activity of *masseter* and *temporalis* muscles at maximum physiological load are an important discriminator of the level of functional adjustment to newly fitted complete dentures. The dynamics of this indicator is featured by marked oscillation in relation to initial values with a tendency to reestablish stability after week 20 from the baseline.

## Figures and Tables

**Table 1:** Comparison of gender, age, and nonexperienced/experienced group.

	Gender		Nonexperienced/experienced group with complete dentures	
	Female	Males	Nonexperienced	Experienced
*N*	42	46	45	43
*X*	54.6	55.7	52.7	57.8
SD	5.4	6.1	5.7	4.1
*X*, max	66	68	65	68
*X*, min	42	44	42	49

**Table 2:** The amplitude dynamics of the planimetric indicators of bioelectric activity of m. masseter (*μ*V) during maximum voluntary tooth compression at the intercuspidal position.

Measurement	Gender				Experience				Total	
	F		M		Without		With			
	DS	NDS	DS	NDS	DS	NDS	DS	NDS	DS	NDS
*N*	42	42	46	46	45	45	43	43	88	88
1x	418	287	509	511	463	420	467	387	465	404
SD	21.3	14.4	27.6	18.7	28.8	27.5	22.3	19.2	18.2	16.9
2x	502	318	594	512	527	448	573	389	550	419
SD	34.9	14.1	17.2	15.2	28.5	20.9	26.3	19.7	19.5	14.7
3x	634	231	586	449	613	387	605	301	609	345
SD	19.9	12.9	17.3	19.1	19.3	23.2	18.4	21.8	13.3	16.5
4x	514	433	537	428	515	417	537	444	526	430
SD	23.6	21.9	27.4	14.7	28.4	16.7	22.6	19.9	18.1	13.0
5x	511	391	747	588	629	496	639	492	634	496
SD	25.0	24.7	32.5	21.6	36.4	26.5	32.0	28.6	24.2	19.4
6x	607	366	734	535	657	448	690	460	673	454
SD	25.9	20.4	27.3	20.4	29.5	25.8	26.9	22.0	20.0	16.9

**Table 3:** The amplitude dynamics of the planimetric indicators of bioelectric activity of m. temporalis during maximum voluntary tooth compression at the intercuspidal position.

Measurement	Gender				Experience				Total	
	F		M		Without		With			
	DS	NDS	DS	NDS	DS	NDS	DS	NDS	DS	NDS
*N*	42	42	46	46	45	45	43	43	88	88
1x	395	342	726	551	612	511	522	389	568	451
SD	22.1	25.7	43.8	26.2	46.7	31.5	38.8	26.0	30.7	21.4
2x	371	264	745	516	609	433	522	356	566	396
SD	16.9	13.8	37.2	33.3	41.6	35.3	39.4	28.1	28.9	22.9
3x	577	374	633	445	641	451	570	369	606	411
SD	22.4	26.1	31.3	25.5	31.6	26.9	21.6	24.0	19.6	18.5
4x	430	366	522	424	496	428	459	363	478	396
SD	13.9	23.1	14.6	17.0	16.1	21.2	15.3	18.5	11.2	14.4
5x	345	348	698	453	539	428	520	377	530	403
SD	8.1	15.4	28.6	19.8	36.1	21.3	32.7	16.8	24.3	13.8
6x	384	354	758	517	596	463	562	415	579	440
SD	9.5	23.4	36.9	21.7	40.6	27.1	39.0	23.6	28.1	18.1

**Table 4:** The values of the sum of the surfaces of the planimetric indicators of the bioelectric activity of m. masseter and m. temporalis.

Measurement	Gender				Experience				Total	
	F		M		Without		With			
	DS	NDS	DS	NDS	DS	NDS	DS	NDS	DS	NDS
*N*	42	42	46	46	45	45	43	43	88	88
1x	65	60	145	103	114	86	100	80	107	83
SD	3.0	2.5	6.8	4.0	9.2	5.2	6.6	4.1	5.7	3.3
2x	136	67	105	153	95	149	78	151	164	87
SD	4.1	2.8	6.3	4.6	6.5	5.3	4.8	3.8	4.1	3.4
3x	191	62	139	81	164	77	165	67	164	72
SD	6.6	2.8	4.0	3.4	6.5	3.5	6.8	3.3	4.7	2.5
4x	132	77	143	100	142	89	134	138	89	100
SD	4.2	2.8	6.2	5.2	6.3	4.8	4.2	4.4	3.8	3.2
5x	127	86	175	110	155	102	149	95	152	99
SD	4.3	3.5	5.7	5.9	7.0	5.9	5.3	4.5	4.4	3.7
6x	157	112	163	104	159	104	161	113	160	108
SD	5.8	6.6	5.2	5.4	5.9	5.9	5.1	6.1	3.9	4.2
